# Rare Extra‐Nasopharyngeal Angiofibroma of the Anterior Nasal Septum Associated With Deviated Nasal Septum: A Case Report

**DOI:** 10.1002/ccr3.70204

**Published:** 2025-02-12

**Authors:** Komal Basharat, Sana Rehman, Faizan Fazal, Abdur Rehman, Javed Iqbal, Shahzaib Maqbool, Imran Khan

**Affiliations:** ^1^ Rawalpindi Medical University Rawalpindi Pakistan; ^2^ Department of ENT and Head and Neck Surgery Benazir Bhutto Hospital Rawalpindi Pakistan; ^3^ Nursing Department, Communicable Diseases Center Hamad Medical Corporation Doha Qatar; ^4^ NYC Health+ Hospital/Woodhull Brooklyn Network New York New York USA

**Keywords:** angiofibroma, nasal obstruction, nasal septum, sinonasal tumor, wounds and injury

## Abstract

Extra‐nasopharyngeal angiofibroma is a rare entity, with only a few cases reported to date. It usually presents with symptoms resembling those of typical nasopharyngeal angiofibroma, but it arises from a site other than the site of origin of typical nasopharyngeal angiofibroma. A 20‐year‐old male presented with complaints of left‐sided nasal obstruction and epistaxis for 10 days. On examination, a mass was seen attached to the anterior nasal septum. Throat and ear examinations were unremarkable. Blood investigations also came out to be as expected. A deviated nasal septum was seen, which was caused by a nasal trauma to this patient 3 years ago. This mass was removed, and the specimen was sent for histologic examination. Histologic examination of the specimen proved the mass to be extra‐nasopharyngeal angiofibroma. The selection of appropriate surgical techniques and subsequent histologic examinations is extremely important to determine the exact diagnosis in such cases. The case addressed in this case report is extremely important for uncovering another rare case of extra‐nasopharyngeal angiofibroma. This case might also suggest a possible linkage between extra‐nasopharyngeal angiofibroma and a deviated nasal septum.

## Graphical abstract

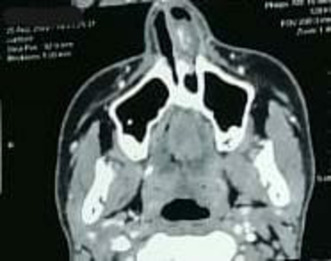

**Summary**

- A rare case of extra‐nasopharyngeal angiofibroma originating from the anterior nasal septum, potentially linked to a prior nasal trauma with deviated nasal septum, was successfully treated surgically.

## 1 Introduction

Juvenile nasopharyngeal angiofibroma is a hypervascular, benign, and locally invasive neoplasm in adolescent males [1, 2, 3]. Extra‐nasopharyngeal angiofibroma, also known as atypical angiofibroma, is a fibrovascular mass that originates from sites other than the typical site of origin of juvenile nasopharyngeal angiofibroma, that is, regions close to the sphenopalatine foramen or pterygoid plates [1, 2, 3, 4, 5, 6]. Extra‐nasopharyngeal angiofibroma differs from the typical nasopharyngeal angiofibroma in various characteristics, including the age of occurrence, gender, pathogenesis, vascularity, etiology, clinical features, and radiographic features [2, 3, 4, 5, 6]. These significant differences from the typical one can be attributed to their atypical site of origin [3]. Extra‐nasopharyngeal angiofibroma originating from the anterior nasal septum and associated with the deviated nasal septum is a rare variant, with only a few cases reported in the literature [7, 8].

Here, we present a case of an extra‐nasopharyngeal angiofibroma originating from the anterior part of the nasal septum and a simultaneous presence of a deviated nasal septum.

## 2 Case Presentation

A 20‐year‐old male presented in the hospital's outpatient department with complaints of left‐sided nasal obstruction and epistaxis for the past 10 days.

**FIGURE 1:**
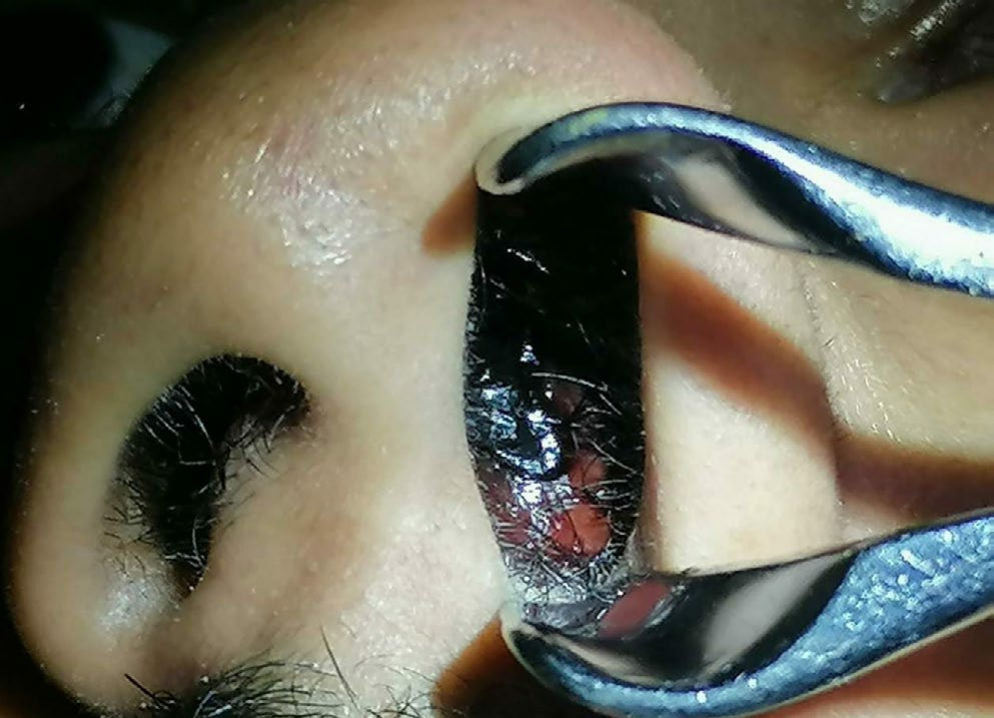
A reddish mass is seen in the left nasal cavity.

The patient noticed a reddish swelling on the left side of the anterior nasal septum in his left nostril. On taking further history, the patient revealed that the swelling appeared 10 days ago from the anterior nasal septum and gradually increased in size over 10 days, subsequently causing worsening left‐sided nasal obstruction. The swelling was reddish‐black. The patient had two to three episodes of epistaxis per day. It contained fresh blood. Bleeding was initially profuse and then later came in the form of drops. There were no associated complaints of pain, anosmia, hyposmia, earache, ear discharge, or sore throat. There was no history of any bleeding disorder. The patient did have a history of nasal trauma due to a frontal blow to the nose 3 years ago. The deviated nasal septum, possibly a consequence of the frontal blow injury, went unnoticed for 2 years until the appearance of a septal mass, which subsequently caused a left‐sided nasal obstruction. Anterior rhinoscopy of the patient revealed reddish swelling filling the left nasal cavity and a left deviated nasal septum, as seen in Figure 1.

**FIGURE 2:**
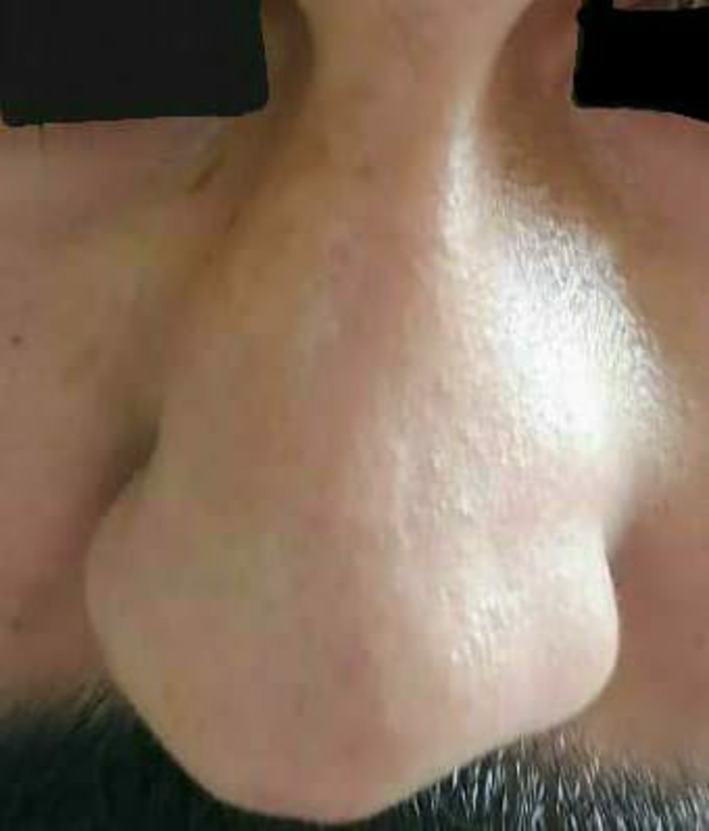
Deviated nasal septum seen on external examination.

An examination of the patient showed a grade 2 external nasal deformity, as seen in Figure 2. A bulge in the left alar region was also observed.

Posterior rhinoscopy was unremarkable. The throat and ear examinations were normal. Cottle's test was positive on the left side. The soft tissue lesion was initially diagnosed as a bleeding nasal polyp.

## 3 Methods

**FIGURE 3:**
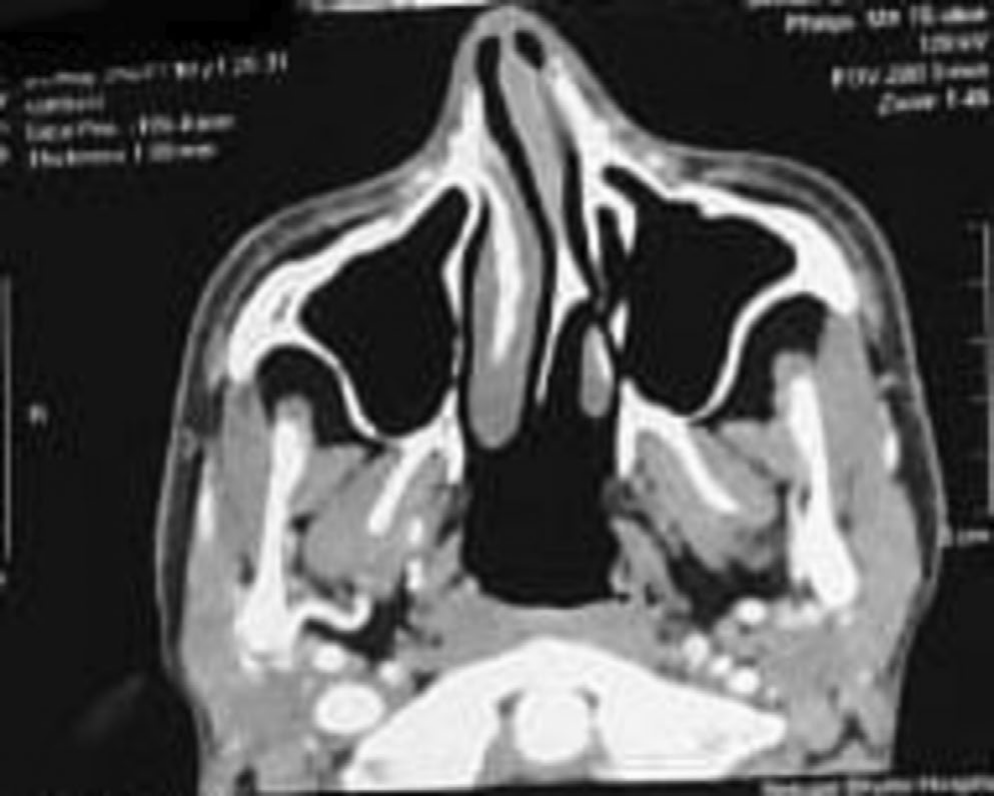
A CT scan of the nose and paranasal sinuses shows a deviated nasal septum. The CT scan further reveals compensatory inferior turbinate hypertrophy on the right side and mild bilateral ethmoidal sinusitis.

A nasal endoscopy was performed to rule out antrochoanal polyp. Bleeding polyp, septal hematoma, and angiofibroma were included in the differential diagnosis. Investigations were carried out to assist in the accurate diagnosis of the patient. Blood coagulation tests, renal function tests, and blood CP were within normal limits. Alkaline phosphatase was slightly raised at 119.2 U/L. A contrast‐enhanced computerized tomography (CT) scan of the paranasal sinuses was performed, which showed a deviated nasal septum with convexity toward the left nasal cavity, as seen in Figure 3.

**FIGURE 4:**
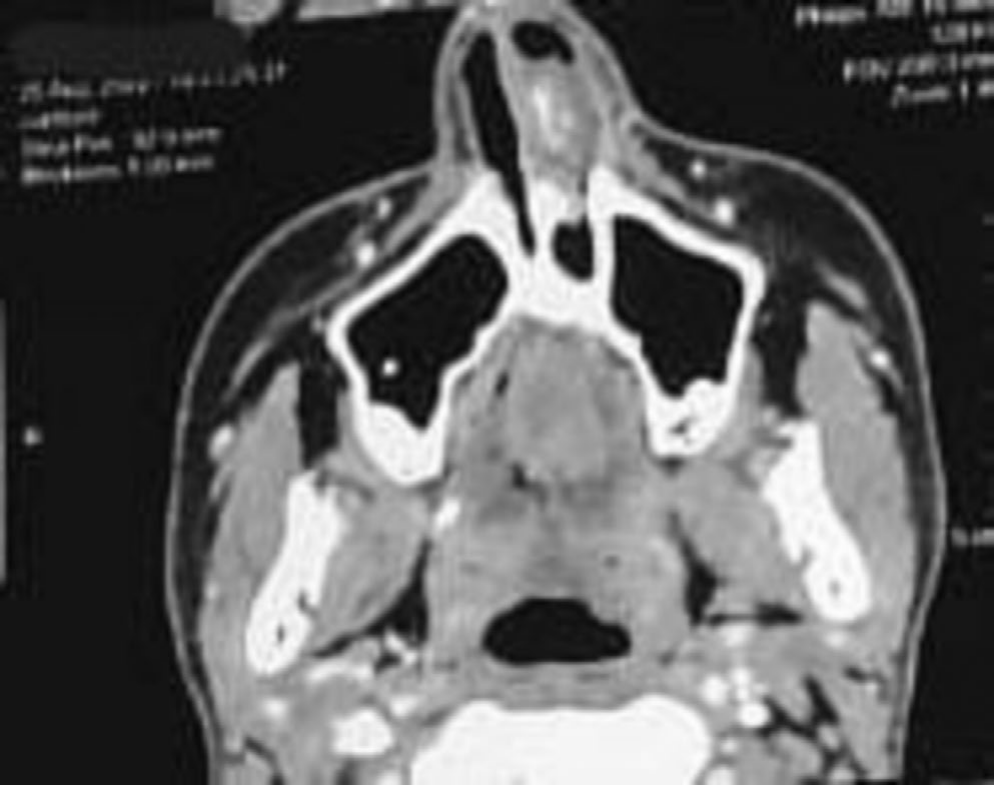
A nose and paranasal sinuses CT scan shows a mass in the left nasal cavity.

A CT scan further revealed compensatory inferior turbinate hypertrophy on the right side, mild bilateral ethmoidal sinusitis, and a 2.1 cm × 1.3 cm × 2.1 cm soft tissue lesion originating from the left side of the anterior nasal septum, as seen in Figure 4.

**FIGURE 5:**
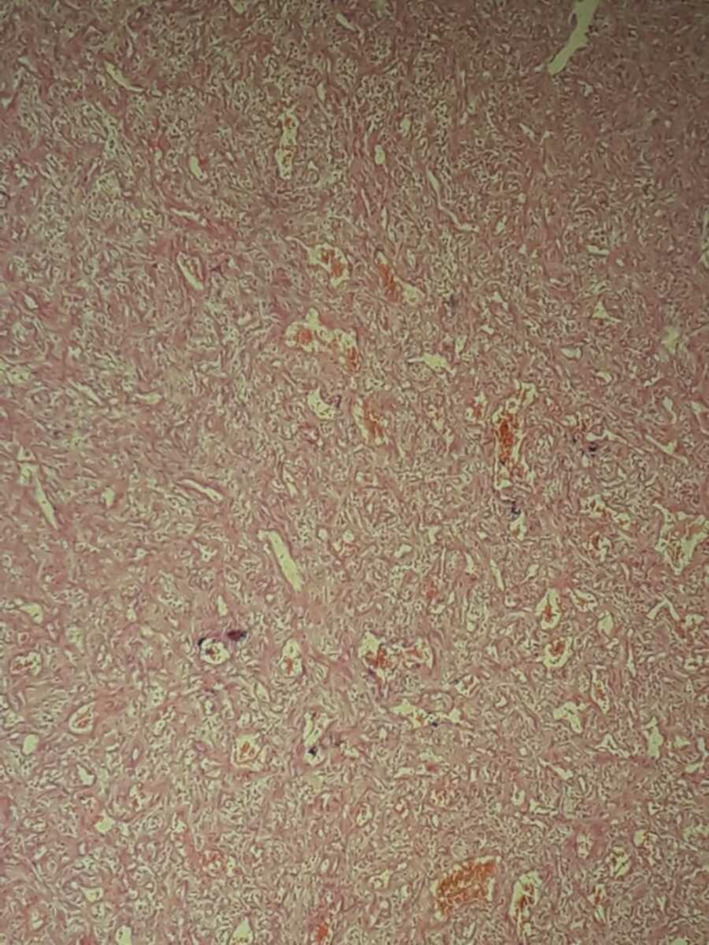
Histopathology reveals pieces of fibrocollagenous tissue consisting of spindle‐shaped fibroblasts and vessels after staining with hematoxylin and eosin under 20× magnification.

Preoperative biopsy was not taken because of the risk of hemorrhage. It was planned to remove the soft tissue lesion seen on contrast CT. The mass was adherent to the septal cartilage. Under general anesthesia and local anesthesia (i.e., Lidocaine HCl 2% with epinephrine (1:100,000) injection USP administered in the nasal mucosa), the periosteum was elevated, and the nasal mass was surgically removed, followed by bipolar cautery. Anterior nasal packing was done to stop bleeding from the septum. Intraoperative frozen section analysis was not done, as this was not available at our hospital. Postoperative recovery was uneventful, and the anterior nasal pack was removed after 48 h. The removed tissue was sent for histopathology to confirm the diagnosis. The histopathology report revealed pieces of fibrocollagenous tissue consisting of spindle‐shaped fibroblasts and vessels, as seen in Figure 5. No evidence of malignancy was seen.

## 4 Conclusion and Results

Histopathology findings were consistent with the diagnosis of nasopharyngeal angiofibroma. However, due to its atypical location, a definitive diagnosis of extra‐nasopharyngeal angiofibroma was made. The surgeons chose to leave the deviated nasal septum untreated because it was insignificant in terms of causing symptoms to the patient.

Extra‐nasopharyngeal angiofibroma originating from the anterior nasal septum is a scarce clinical entity, with only a few cases reported in the literature. Extra‐nasopharyngeal angiofibroma is locally invasive, can grow in size, and can cause debilitating symptoms of profuse epistaxis and nasal obstruction. Not much is known about extra‐nasopharyngeal angiofibroma because of its rare occurrence. Thus, extra‐nasopharyngeal angiofibroma requires special attention while their investigations and treatment are planned. Obtaining a histopathologic report is necessary to make a definitive diagnosis of extra‐nasopharyngeal angiofibroma. Fibrocollagenous tissue consisting of spindle‐shaped fibroblasts and vessels is the histology in extra‐nasopharyngeal angiofibroma. Extra‐nasopharyngeal angiofibroma has not been known to recur after surgical removal. Thus, selecting proper surgical techniques is vital in treating this angiofibroma. A deviated nasal septum might be linked to the subsequent development of extra‐nasopharyngeal angiofibroma.

## 5 Discussion

Typical juvenile nasopharyngeal angiofibroma constitutes < 0.05% of all head and neck neoplasms [2, 3, 9]. It arises from the vicinity of the sphenopalatine foramen or pterygoid plates [1, 2, 3, 4, 5, 6].

On the contrary, extra‐nasopharyngeal angiofibroma is a rare clinical entity originating from sites other than the typical site of origin of nasopharyngeal angiofibroma. The nasal septum, maxilla, inferior turbinate, middle turbinate, ethmoid sinus, sphenoid sinus, oral cavity, oropharynx, ear, larynx, trachea, middle cranial fossa, and infratemporal fossa are the affected sites. A case of oropharyngeal (posterior tonsillar pillar) extra‐nasopharyngeal angiofibroma was reported in India in a 26‐year‐old male. This patient presented with foreign body sensation and a gradually progressing nasal mass for 1 month. The excision of the mass was carried out under general anesthesia, and there was no recurrence after that [10]. Unlike juvenile nasopharyngeal angiofibroma, which predominantly affects males in their second decade, extra‐nasopharyngeal angiofibroma affects older females [3, 4, 6, 7, 8, 9]. However, in our case, it was presented in a 20‐year‐old male, which is inconsistent with the usual behavior of extra‐nasopharyngeal angiofibroma. These lesions are less vascular than the juvenile nasopharyngeal angiofibroma [3, 7, 9, 11]. Theories encompassing embryologic development, genetics, and hormonal changes causing such pathologies have been suggested.

However, the exact reason behind this unusual presentation of extra‐nasopharyngeal angiofibroma is unknown [1, 11]. The hormonal theory has been rejected on account of its predominance in the female gender, suggesting that its growth is not influenced by the presence or absence of testosterone [11]. On the contrary, the growth of typical nasopharyngeal angiofibroma is driven by testosterone, which is the reason behind the predominance of typical nasopharyngeal angiofibroma in adolescent males [6]. The developmental theory does point toward the anomalous anterior expansion of fascia basalis beyond its usual site (i.e., the posterior portion of the vomer and ethmoid bone), forming nasal septal angiofibroma [2, 9, 11]. However, this theory explains the pathogenesis of those arising from the osseocartilaginous junction but fails to explain those arising from the anterior part of the nasal septum [9]. Another theory suggests that persistent vascular tissue mimicking turbinate tissue in the nasal septum during embryologic development gives rise to extra‐nasopharyngeal angiofibroma later in life [9]. Extra‐nasopharyngeal angiofibroma of the nasal septum arises from either of the three locations, that is, anterior septum, posterior septum, or osseocartilaginous junction [7]. In our case report, extra‐nasopharyngeal angiofibroma's site of origin is the anterior part of the nasal septum. Septal involvement may initially give a false impression of a bleeding nasal polypus [8]. In our case, it was initially diagnosed as a bleeding nasal polypus.

Later, the histopathology report revealed it to be an angiofibroma in an atypical location; hence, it was labeled as an extra‐nasopharyngeal angiofibroma. To properly assess the tumor's size, blood supply, and major blood vessels, preoperative tests for extra‐nasopharyngeal angiofibroma include nasal endoscopy, CT, MRI, and angiography. Pre‐op embolization is carried out in nasopharyngeal angiofibroma but is not needed in extra‐nasopharyngeal angiofibroma as it is less vascular [12]. Contrast‐enhanced CT and MRI are the investigations of choice, as suggested by most authors; however, the histopathologic evaluation can make the final diagnosis [2, 6]. A Holman Miller sign on CT, that is, anterior arching of the posterior wall of the maxillary sinus [13], is a pathognomonic radiographic finding in juvenile nasopharyngeal angiofibroma [14]. In this case, the Holman Miller sign was absent due to its atypical location. One author suggested that immunohistochemistry should be carried out in addition to other investigations [6]. Another author suggested selective angiography as a beneficial confirmatory test for diagnosis [3]. The histopathologic findings of extra‐nasopharyngeal angiofibroma and juvenile nasopharyngeal angiofibroma are almost identical and reveal them to be fibrovascular masses composed of stellate‐shaped fibroblasts and vessels [6]. The two clinical entities also differ from each other concerning vascularity, with extra‐nasopharyngeal angiofibroma being less vascular than juvenile nasopharyngeal angiofibroma; however, preoperative biopsy to reach a clinical diagnosis is contraindicated in both cases due to the fear of extensive hemorrhage [3, 9]. Surgical removal followed by histopathologic evaluation is done to treat and accurately diagnose the disease [1, 3, 5, 9]. A myriad of surgical approaches has been undertaken by surgeons to surgically remove the tumors depending upon their site of origin, including lateral rhinotomy, sub‐labial approach with carbon dioxide laser, and endoscopic techniques [5]. In extra‐nasopharyngeal angiofibroma, the endoscopic and endonasal approach should be used. If there is excessive bleeding, it requires an external approach. Sclerosing, radiation, preoperative hormone therapy, chemotherapy, and vascular ligation are some other treatment options [15].

Juvenile nasopharyngeal angiofibroma does not allow easier access during surgical removal; therefore, its treatment has been associated with poor outcomes, with most of the cases reappearing within 1 year. However, the reappearance of extra‐nasopharyngeal angiofibroma is rare [16]. Only four individuals in a systematic study experienced extra‐nasopharyngeal angiofibroma recurrences compared to nasopharyngeal angiofibroma; all of these patients did so within a year following diagnosis, and only one patient in a juvenile case did so as soon as 2 weeks following surgery [17]. Follow‐up of our case for up to 1 year showed no reappearance of extra‐nasopharyngeal angiofibroma as well.

Only a few cases of extra‐nasopharyngeal angiofibroma have been seen worldwide to date. Thus, any case of extra‐nasopharyngeal angiofibroma is sporadic in itself. Of all cases of extra‐nasopharyngeal angiofibroma seen to date, a deviated nasal septum has not been seen in any of the cases. Thus, a deviated nasal septum and extra‐nasopharyngeal angiofibroma make it even rarer.

## Author Contributions

**Komal Basharat:** conceptualization, writing – original draft. **Sana Rehman:** conceptualization, data curation, investigation. **Faizan Fazal:** conceptualization, software, writing – original draft. **Abdur Rehman:** methodology, software, writing – review and editing. **Javed Iqbal:** funding acquisition, resources, supervision. **Shahzaib Maqbool:** methodology, software, writing – review and editing. **Imran Khan:** funding acquisition, software, writing – review and editing.

## Ethics Statement

Informed consent was taken from the patient, and ethical considerations were discussed.

## Consent

Written informed consent was obtained from the patient for the publication of the patient's clinical details and clinical images.

## Conflicts of Interest

The authors declare no conflicts of interest.

## Data Availability

The data regarding the case is available upon reasonable request.
